# The prevalence and predictors of fear of childbirth among pregnant Chinese women: a hierarchical regression analysis

**DOI:** 10.1186/s12884-021-04123-7

**Published:** 2021-09-22

**Authors:** Jingui Huang, Jing Huang, Yan Li, Bizhen Liao

**Affiliations:** 1 Present Address: Department of Obstetrics, The First Affiliated Hospital of Chongqing Medical University, No. 1, Youyilu Street, Yuzhong District, Chongqing, 400016 China; 2 Department of Endocrinology, The First Affiliated Hospital of Chongqing Medical University, Chongqing, 400016 China

**Keywords:** Fear of childbirth, Resilience, Childbirth self-efficacy, Prevalence, Predictors

## Abstract

**Background:**

Fear of childbirth (FOC) occurs before, during and after pregnancy and is harmful to both the pregnant woman and the fetus. Identifying the prevalence and predictors of FOC can help us generate strategies for alleviating women’s FOC.

**Methods:**

A cross-sectional study was conducted among a convenience sample of 646 pregnant women receiving antenatal care at a subordinate hospital of a university in China. Data were collected using a basic information form, the Childbirth Attitude Questionnaire, the Childbirth Self-Efficacy Inventory, and the 10-item Connor-Davidson Resilience Scale. The minimum and maximum total scores of the Childbirth Attitude Questionnaire are 16 and 64, respectively, with higher scores reflecting a greater degree of FOC. We conducted hierarchical regression analysis to explore the predictors of FOC and used a structural equation model to further examine the direct and indirect associations between FOC, resilience and childbirth self-efficacy.

**Results:**

The total prevalence of FOC was 67.1%. The percentages of women with mild (score of 28–39), moderate (40–51), and severe FOC (52–64) were 45.4, 19.5, and 2.2%, respectively. The average score on the Childbirth Attitude Questionnaire was 32.49, indicating mild FOC. The final regression analysis revealed six variables predicting FOC that explained 64.5% of the variance in FOC: age, gestational age, parity, spousal support, resilience, and childbirth self-efficacy. Furthermore, childbirth self-efficacy mediated the relationship between resilience and FOC, and the mediation effect rate was 53.5%.

**Conclusions:**

A high prevalence of FOC among pregnant Chinese women was found in this study. Age, gestational age, parity, spousal support, resilience, and childbirth self-efficacy were predictors of FOC. It is suggested that healthcare professionals should pay close attention to FOC and implement targeted interventions in accordance with these predictors, especially resilience and childbirth self-efficacy.

## Background

Fear of childbirth (FOC) is a health issue for a pregnant woman that is similar to an anxiety disorder or a phobic fear and involves physical complications, nightmares and concentration problems [1]. An increasing body of evidence suggests that FOC can affect a woman’s relationship with the baby, her partner and her family [2, 3], and often results in more frequent requests for epidural analgesia and cesarean section [4–7]. Moreover, FOC is also related to posttraumatic stress disorder [8, 9] and a longer duration of labor [7, 10]. FOC is a common psychological problem for pregnant women. Approximately 20% of gravidas experience FOC, according to existing studies [11, 12]. A meta-analysis reported a 14% pooled prevalence of FOC, but with significant heterogeneity [13]. It is normal for FOC to differ across countries considering that birth is an omnifarious experience.

Prior studies indicate that FOC is caused by multiple factors, including obstetric, sociodemographic and psychological variables. Research on the effects of obstetric factors on FOC has shown that parity [14], planned pregnancy [15] and gestational week [11] affect FOC. However, no connection was found between FOC and conception type [16, 17]. In terms of sociodemographic factors, previous studies found that age [18, 19], educational level [20, 21], income level [19], and employment status [20, 21] are connected with FOC. Moreover, a lack of social or spousal support is connected to an increased probability of FOC [22, 23]. Among psychological issues, childbirth self-efficacy may be linked to FOC. The higher the childbirth self-efficacy women reported was, the lower their level of FOC [24, 25].

It is worth noting that resilience, proposed and developed by positive psychology and representing one’s capacity for survival and adjustment after experiencing serious traumatic events [26], can help people accommodate, handle or pass through adversity based on a self-regulating psychological mechanism [27] and recover from disasters or maintain their psychological health [28–30]. As FOC is a negative emotional experience, we speculate that resilience may have an impact on it. Additionally, research has stated that resilience influences self-efficacy [31, 32], and self-efficacy has been examined as a mediator of mental health outcomes [33]. Hence, we hypothesized that childbirth self-efficacy and resilience would have a direct effect on FOC and that resilience would have an indirect effect on FOC via childbirth self-efficacy.

This is the first study to examine the impact of resilience on FOC and the relationships among FOC, childbirth self-efficacy and resilience in pregnant women. In addition, only two studies conducted by Chinese scholars have examined the factors affecting FOC but without discussing the extent to which these variables predict FOC [20, 34]. Taking all the reasons mentioned above into consideration, the main objective of this study is to identify the levels of FOC and evaluate the predictive factors among pregnant Chinese women and to explore the interrelationships among FOC, childbirth self-efficacy, and resilience.

## Methods

### Design and participants

A cross-sectional survey was conducted in the First Affiliated Hospital of Chongqing Medical University. The study was performed in accordance with the Declaration of Helsinki and was approved by the local Ethics Committee. Chongqing is a municipality located in southwestern China and has a population of approximately 31 million. The birth rate at the study hospital is more than 9000 babies per year. Pregnant women who established a health record (gestational week ≥ 11) and attended routine prenatal examinations at the obstetrics clinic at the time of the research period were recruited. The inclusion criteria were Chinese pregnant women with a singleton pregnancy who were 18 years old or older, had no pregnancy complications, and had no previous cesarean section or psychiatric disorders. The exclusion criteria were women who had signs of cesarean section or declined to participate in the survey. Three well-trained researchers collected the data from August to December 2020 via a face-to-face survey. After obtaining written informed consent from participants, the researchers distributed the anonymous questionnaires and instructed them on how to fill them out. Excluding 39 respondents because of incorrect or incomplete responses and refusal, we analyzed the responses of 646 pregnant women.

### Measures

#### Basic information form

The basic information form included eleven questions on respondents’ age, education, occupation, marital status, residence, family per capita monthly income (RMB, renminbi, Chinese yuan), gestational age, parity, planning pregnancy, conception type, and spousal support.

#### Fear of childbirth

Consisting of 16 items, the Childbirth Attitude Questionnaire(CAQ) was developed to measure FOC [35]. Responses are given on a four-point Likert scale, and scores range from 16 to 64, with higher scores indicating higher FOC. The scale included four domains: fear of fetal health; fear of losing control during childbirth; fear of childbirth pain; fear of medical intervention and the hospital environment. CAQ total scores were categorized as none (16–27), mild (28–39), moderate (40–51) and severe (52–64). Wei wand her colleagues translated the scale into Chinese, and this scale has good reliability (Cronbach’s α = 0.91) and validity (content validity index (CVI) = 0.924) [36]. Cronbach’s α was 0.92, and the CVI was 0.930 in this study. Cronbach’s α coefficient represents internal consistency reliability, and an α coefficient ≥ 0.70 indicates acceptable reliability [37].

#### Childbirth self-efficacy

The short form of 32-item Chinese Childbirth Self-Efficacy Inventory (CBSEI-C32) was used to measure childbirth self-efficacy. The Outcome Expectancy Subscale (OE-16) and Efficacy Expectancy Subscale (EE-16) make up the CBSEI-C32 [38]. Each item is answered on a ten-point Likert scale ranging from 1 to 10. Total scores range from 32 to 320, and the higher scores are, the higher the self-efficacy. The Chinese version of the CBSEI-C32 has high internal consistency (Cronbach’s α = 0.96) and test–retest reliability (intraclass correlation coefficient = 0.88), and significant Pearson’s correlations with measures of general sense of perceived self-efficacy (*r* = 0.32, *P* < 0.01) and anxiety (*r* = -0.21, *P* < 0.01) indicate its excellent construct validity [39]. The Cronbach’s alpha for each subscale was 0.96 and 0.97, and the CVI was 0.962 in this study.

#### Resilience

To measure resilience in pregnant Chinese women, we used the 10-item Connor-Davidson Resilience Scale (CD-RISC-10) in this study. Campbell-Sills and Stein created the original English version of the CD-RISC-10 [40]. Then, the scale was translated into Chinese and used to measure resilience in Chinese earthquake victims by Wang and his colleagues [41]. Responses are given on a four-point Likert scale, ranging from 0(“never”) to 4 (“nearly always”), with higher total scores representing better levels of resilience. The Chinese CD-RISC-10 has a Cronbach’s alpha of 0.91, and significant Pearson’s correlations with measures of posttraumatic stress disorder indicate its satisfactory construct validity (*r* = -0.53, *P* < 0.01) [41]. In this study, the Cronbach’s alpha was 0.91, and the CVI was 0.925.

### Statistical analysis

The mean and standard deviation (SD) were used to describe continuous variables and frequencies with percentages were used to summarize categorical variables. We performed an independent t-test and 1-way analysis of variance (ANOVA) to compare the CAQ scores between different characteristics, and Pearson correlation analyses were used to test the relationships between FOC and self-efficacy and resilience. If the above variables had a* P* value < 0.05 in a t-test/ANOVA or Pearson correlation analysis, they were retained in the hierarchical regression analysis model. Cook’s distances (< 1.0) were computed to identify influential cases and outliers. The Cook’s distances varied between 0.0000 and 0.08679 in this study. The Durbin–Watson (DW) statistic was used to test the independence of error terms and the sequential correlation of adjacent errors. This statistic can range from 0 to 4, with a value of 2 indicating that the residuals are uncorrelated. The DW value was 1.900 in our study. The variance inflation factor (VIF) was applied to diagnose the possibility of multicollinearity among all the explanatory variables. A VIF less than 5 indicates that there is no serious multicollinearity. All the VIF values were < 5 in this study. A *P* value < 0.05 was considered statistically significant. Data were recorded using EpiData version 3.1 after checking for completeness, and analyses were conducted using IBM SPSS Statistics version 25.

We performed structural equation modeling to analyze the mediation model. A model was established with FOC as the dependent variable, resilience as the independent variable, and childbirth self-efficacy as the mediating variable. Maximum likelihood estimation was employed as a global test of models. The bootstrapping method was used to test the significance of the indirect effect of a mediator. It is believed that an indirect effect is significant at the 0.05 level if the bias corrected 95% confidence interval (CI) from 5000 bootstrap samples does not include 0. Amos 23.0 was used for the modeling. The structural equation model (SEM) was acceptable with the following indexes: *x*^2^/*df* < 3; root mean square error of approximation (RMSEA) < 0.08; goodness of fit index (GFI) and adjusted goodness of fit index (AGFI) values > 0.90; comparative fit index (CFI) and normed fit index (NFI) values > 0.90; and incremental fit index (IFI) and relative fit index (RFI) values > 0.90 [42].

## Results

### Description of participants’ basic characteristics and their correlations with FOC

Table 1 shows the sample’s sociodemographic and obstetric characteristics and their associations with the CAQ scores. The age range was 18 to 42 years, with a mean age of 28.7 (SD = 3.8). Regarding sociodemographic status, 81.9% of the women had a college education and most participants were employed (82.8%). The clear majority (98.9%) were married and lived in cities (91.5%). Half of the women had a family per capita monthly income of 4000–8000 RMB (50.9%), and the majority of the women had their partner’s full support for their current pregnancy (85.8%). In terms of the main obstetric information, the mean gestational age was 29.3 weeks and 82.4% were nulliparous.

**Table 1:** Participants’ basic characteristics and their correlations with FOC (*n* = 646)

Characteristics	N (%)	CAQ scores (Mean ± SD)	*F* or *t* value	*P* Value
**Age (years)**			3.324	0.019
18–25	105 (16.3)	33.61 ± 9.22		
26–30	346 (53.6)	32.38 ± 8.30		
31–35	156 (24.1)	31.24 ± 8.32		
36–42	39 (6.0)	35.51 ± 10.40		
**Educational**			4.009	0.008
Junior middle school or below	31 (4.8)	34.61 ± 9.28		
Senior middle school or same level	86 (13.3)	32.67 ± 8.25		
University or Junior college	460 (71.2)	32.80 ± 8.56		
Master degree or above	69 (10.7)	29.30 ± 8.82		
**Occupation**			1.590	0.161
Office clerk	251 (38.9)	33.06 ± 8.56		
Executive staff/civil servant	36 (5.6)	32.28 ± 7.63		
Medical, educational and scientific personnel	139 (21.5)	31.13 ± 8.55		
Self-employed	39 (6.0)	32.15 ± 8.63		
Other	70 (10.8)	31.49 ± 8.99		
Unemployed	111 (17.2)	33.75 ± 8.92		
**Marital status**			2.496	0.013
Married	639 (98.9)	32.41 ± 8.60		
Other (Divorced/Separated/ Single)	7 (1.1)	40.57 ± 9.36		
**Residence**			1.483	0.228
Urban	591 (91.5)	32.46 ± 8.62		
Town	36 (5.6)	31.44 ± 8.98		
Rural	19 (2.9)	35.58 ± 8.53		
**Family per capita monthly income (RMB)**			0.015	0.985
< 4000	54 (8.4)	32.31 ± 9.17		
4000–8000	329 (50.9)	32.49 ± 8.56		
> 8000	263 (40.7)	32.54 ± 8.67		
**Gestational age (week)**			4.187	0.016
11–12	61 (9.4)	30.16 ± 9.37		
13–28	162 (25.1)	31.67 ± 8.33		
29–40	423 (65.5)	33.14 ± 8.59		
**Parity**			4.688	0.038
Nullipara	532 (82.4)	33.22 ± 8.68		
Multipara	114 (17.6)	29.11 ± 7.61		
**Pregnancy planning**			5.523	0.000
Yes	431 (66.7)	31.25 ± 8.83		
No	215 (33.3)	34.98 ± 7.69		
**Conception type**			0.171	0.864
Spontaneous fertilization	613 (94.9)	32.51 ± 8.63		
Assisted fertilization	33 (5.1)	32.24 ± 9.05		
**Prenatal spousal support**			19.594	0.000
No support	6 (0.9)	32.49 ± 8.64		
Very few support	8 (1.2)	43.38 ± 11.80		
General support	78 (12.1)	36.81 ± 8.38		
Full support	554 (85.8)	31.58 ± 8.18		

*RMB* Renminbi (Chinese Yuan), *FOC* Fear of childbirth, *CAQ* Childbirth Attitude Questionnaire, *SD* Standard Deviation

In the univariate analysis of the factors related to the CAQ scores, seven factors were significantly related to FOC (*P* < 0.05): age, education, marital status, gestational age, parity, pregnancy planning and spousal support. More detailed information is presented in Table 1.

### FOC levels and the correlations among FOC, self-efficacy, and resilience

The CAQ, self-efficacy, and resilience scale scores are shown in Table 2. Among a total of 646 participants, the prevalence rates of mild, moderate, and severe FOC were 45.4% (*n* = 293), 19.5% (*n* = 126), and 2.2% (*n* = 14), respectively. The mean CAQ score was 32.49 ± 8.64. Table 2 also shows the relationship among FOC, self-efficacy and resilience. Pearson correlation analyses demonstrated that the CAQ scores were inversely related to CBSIE-32 scores, and CD-RISC-10 scores. Specifically, a positive correlation was found between the CBSIE-32 and CD-RISC-10 scores.

**Table 2:** Scores of each scale in pregnant women and Pearson correlation coefficients (*n* = 646)

Scales	Scores (Mean ± SD)	1	2	3
1.CAQ (range: 16–61)	32.49 ± 8.64	1	–	–
2. CBSIE-32(range: 54–320)	201.44 ± 58.69	-0.738**	1	–
OE-16 (range: 26–160)	100.38 ± 30.02	-0.711**	–	–
EE-16 (range: 23–160)	101.05 ± 30.01	-0.732**	–	–
3. CD-RISC-10 (range: 7–40)	26.51 ± 5.83	-0.638**	0.593**	1

*CAQ* Childbirth Attitude Questionnaire, *CBSIE-32* The short form of 32-item Chinese Childbirth Self-Efficacy Inventory, *CD-RISC-10* The 10-item Connor-Davidson Resilience Scale, *OE-16* Outcome Expectancy Subscale, *EE-16* Efficacy Expectancy Subscale, *SD* Standard Deviation

^**^P < 0.01

### The results of hierarchical regression analysis regarding predictors of FOC

A three-step hierarchical regression analysis was performed to identify factors predicting FOC. The independent variables were entered as follows: step 1 included demographic and obstetric variables such as age, educational level, marital status, gestational age, parity, pregnancy planning and spousal support; step 2 included resilience variables; and step 3 included childbirth self-efficacy variables. The results of the regression analysis related to the independent variables predicting FOC are depicted in Table 3.

**Table 3:** Hierarchical regression analysis of variables in predicting FOC (*n* = 646)

Variables	Model 1			Model 2			Model 3		
	B	β	*P*	B	β	*P*	B	β	*P*
(Constant)	37.801	–	< 0.001	58.560	–	< 0.001	62.071	–	< 0.001
**Age** (ref: 36–42)									
31–35	-3.159	-.157	.028	-2.543	-.126	0.025	-1.900	-.094	.045
26–30	-3.877	-.224	.005	-3.919	-.226	< 0.001	-3.029	-.175	.001
18–25	-4.159	-.178	.006	-4.650	-.199	< 0.001	-3.338	-.143	.001
**Educational level** (ref: Junior middle school or below)									
Senior middle school or same level	-2.530	-.100	.126	-.837	-.033	.520	-.157	-.006	.886
University or Junior college	-1.977	-.105	.185	.272	.014	.819	.167	.009	.867
Master degree or above	-4.454	-.159	.011	-1.305	-.047	.346	-1.405	-.037	.368
**Marital status** (ref: Married)									
Other (Divorced/Separated/ Single)	4.823	.214	< 0.001	1.539	.049	.309	1.055	.031	.426
**Gestational age** (ref: 29–40)									
13–28	-1.657	-.083	.027	-1.530	-.077	.009	-1.292	-.065	.009
11–12	-3.230	-.109	.003	-2.001	-.068	.018	-1.486	-.050	.037
**Parity** (ref: Nullipara)									
Multipara	-5.585	-.247	< 0.001	-4.284	-.189	< 0.001	-3.502	-.135	< 0.001
**Pregnancy planning** (ref: Yes)									
No	3.710	.202	< 0.001	2.270	.124	< 0.001	.841	.046	.068
**Spousal support** (ref: Full support)									
General support	4.487	.169	< 0.001	3.423	.129	< 0.001	2.033	.077	.002
Very few support	12.392	.159	< 0.001	8.945	.115	< 0.001	7.263	.092	< 0.001
No support	12.171	.135	< 0.001	9.766	.108	< 0.001	8.340	.093	< 0.001
**CD-RISC-10**^a^	–	–	–	-.855	-.577	< 0.001	-.453	-.305	< 0.001
**CBSIE-32**^a^	–	–	–	–	–	–	-.073	-.495	< 0.001
*R*^2^			0.189			0.494			0.645
*R*^2^ change			0.189			0.305			0.151
F			11.709			43.888			75.289
Sig. of the model			< 0.001			< 0.001			< 0.001

*B* Unstandardized coefficients, *β* Standardized coefficients, *ref* Reference, *Sig.* Significance, *CD-RISC-10* The 10-item Connor-Davidson Resilience Scale, *CBSIE-32* The short form of 32-item Chinese Childbirth Self-Efficacy Inventory, *FOC* Fear of childbirth

^a^Continuous variable

In the first model, obstetric and sociodemographic variables significantly explained 18.9% of the variance in FOC (*F* = 11.709, *P* < 0.01). In the second model, the model significantly explained 49.4% of the variance in FOC with the inclusion of the CD-RISC-10 score (*F* = 43.888, *P* < 0.01). In the third model, the addition of childbirth self-efficacy led to an improvement in the model, with significant changes in R^2^ of 15.1% (*F* = 75.289, *P* < 0.01). Overall, the final model explained 64.5% of the variance in FOC and revealed six variables that contributed significantly to FOC. Childbirth self-efficacy was the strongest predictor of FOC, followed by resilience. Regarding sociodemographic variables, advanced age, late pregnancy, being nulliparous, and poor spousal support were predictors of a higher degree of FOC.

### Mediating effect of childbirth self-efficacy on the relation between resilience and FOC

**Fig. 1:**
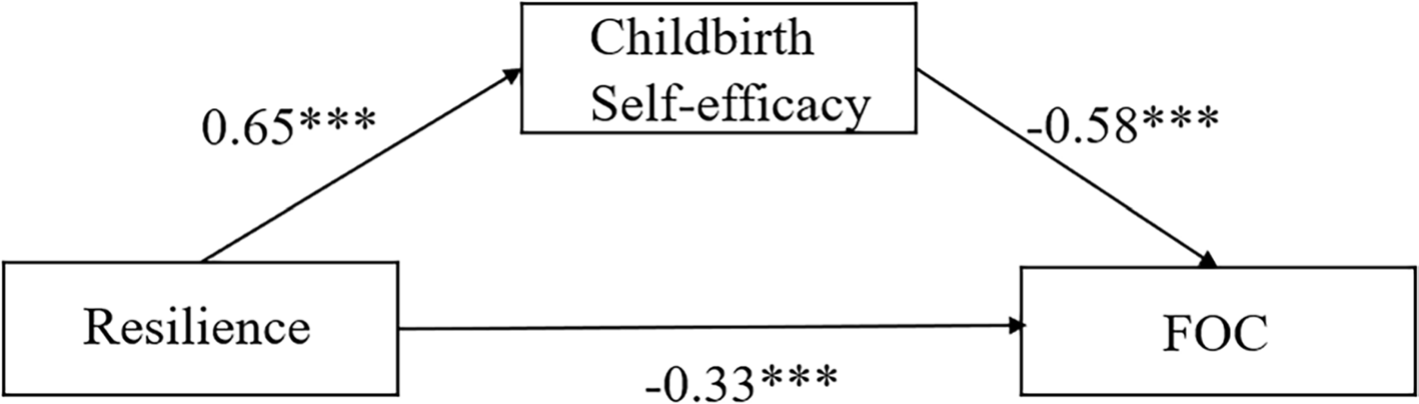
The model of the mediating role of childbirth self-efficacy on the association between resilience and FOC (*n* = 646). Note: ***P < 0.001; FOC: fear of childbirth

Figure 1 depicts the mediation models of childbirth self-efficacy and the standardized coefficients for each variable. The SEM showed significant regression and correlation paths, with all the path coefficients being statistically significant at the level of *P* < 0.05. The fit indices for the model were acceptable: *x*^2^/*df* = 2.645, RMSEA = 0.051, GFI = 0.951, AGFI = 0.932, CFI = 0.976, NFI = 0.962, IFI = 0.976, and RFI = 0.953.

According to the model, FOC was significantly predicted by resilience and childbirth self-efficacy. The standardized direct effect value of childbirth self-efficacy on FOC was -0.58(*P* < 0.001), and the standardized direct effect value of resilience on FOC was -0.33 (*P* < 0.001). Resilience significantly predicted childbirth self-efficacy, and the standardized direct effect of resilience on self-efficacy was 0.65 (*P* < 0.001). The bootstrapped 95% CI did not include 0 (-0.438 ~ -0.316, *P* = 0.000), confirming that the indirect effect of resilience on FOC through childbirth self-efficacy was significant. The standardized indirect effect of resilience on FOC through self-efficacy was -0.38. The standardized total effect of resilience on FOC was -0.71. Therefore, indirect effects account for 53.5% of the total effect.

## Discussion

### The prevalence of FOC

The mean CAQ score in our study was 32.49. Comparing this mean score with those from other results, it is slightly higher than that in studies from China that used the same assessment tool (32.20 and 31.30) [20, 34]. Our data showed that FOC occurred in 67% of pregnancies, and 2.2% of participants experienced severe FOC. Several studies reported the following rates of severe FOC: 5% in Australia, 5.3% in Ireland, 6.1% in Iran, 8% in Kenya, 20.8% in Turkey and 24.5% in Ethiopia [43–48]. It is difficult to compare the incidence of FOC across countries due to differences in the measures and definitions used. However, one conclusion we can draw is that FOC is a prevalent psychological problem among pregnant Chinese women, and most of them were experienced mild or moderate FOC. It is time for healthcare professionals to understand, recognize and intervene in FOC.

### Demographic and obstetric factors predicting FOC

We performed hierarchical regression analysis to confirm the correlations of sociodemographic, obstetric, and other characteristics with FOC. The best-fit regression model revealed six variables that explained 64.5% of the variance in the CAQ score. Of the sociodemographic factors, advanced age was found to predict FOC, in agreement with a previous study from Finland [19]. This may have something to do with women’s belief that advanced age makes them unfit to give birth. However, Laursen et al. [18] demonstrated that young women < 20 years reported intense FOC in a study from Denmark. Very young women are worried that they will not be able to take care of their child appropriately [49]. In addition, poor partner support was correlated with FOC. Similarly, previous studies showed that receiving a low level of support from one’s husband increased the probability of FOC [17, 22, 45]. In Turkey, Çıtak et al. [50], in contrast to our study, found that spousal support did not predict FOC. Reproductive health at birth is considered a woman’s responsibility in Turkey, so the expectation of partner support is low.

Among the obstetric features, gestational age and parity are significant predictors of FOC. As the gestational week increased, a higher level of FOC was more likely to be reported, consistent with a previous study [51]. As in other studies [51, 52], multiparas had lower levels of FOC than primiparas. This is reasonable because multiparas have more experience and information about the whole delivery process [53]. However, previous work by Räisänen et al. stated that multiparous women had a higher risk of experiencing FOC [19], in which case FOC was usually related to a previous traumatic or negative childbirth experience [54, 55].

From the discussion above, we can clearly see that different studies report conflicting results regarding the association between the FOC and sociodemographic and obstetric factors. In the current study, however, the sample sources are relatively limited in terms of achieving adequate power to explore the inconsistencies surrounding this issue, and more specific research is necessary to examine their association. However, it is of great importance to design antenatal educational programs targeting for different demographic and obstetric backgrounds.

### Resilience

When we added resilience to the model in the second step, the model explained 49.4% of the variance in FOC, indicating that resilience plays a significant role in predicting FOC, a finding that has not been reported before. In terms of resilience, an increasing body of evidence suggests that resilience serves as a protective factor for psychological health and overall well-being [56, 57]. Although childbirth is a normal and healthy life experience, pregnant women with FOC may regard it as a challenge and adversity. Resilience helps individuals cope with such adversities and difficulties, so pregnant women with a better level of resilience may manage their emotions successfully, actively use their own psychological qualities to cope with the stress of childbirth, and ultimately reduce fear. In a Chinese study involving 2813 pregnant women, resilience was found to have a significant independent protective effect on prenatal anxiety/depression [58].

To the best of our knowledge, this is the first study to explore the association between resilience and FOC, providing a new perspective for developing related interventions. Health care professionals are advised to focus on resilience interventions and the formulation and implementation of programs to enhance resilience. Interventions including emotional regulation training, cognitive and behavioral therapy to reframe thoughts and refocus on positive emotions, and physical health improvement via exercise, sleep, nutrition, and mindfulness have the potential to enhance resilience [59–61]. Evidence has shown that social support or family support can provide powerful external conditions for the development of resilience [62, 63]. More specifically, higher support obtained from social networks can help pregnant women positively cope with the stressors resulting from pregnancy and childbirth. Suggested measures include encouraging husbands or other family members to provide adequate spiritual and material support for pregnant women and participate in antenatal courses and providing group activities such as peer support groups or pregnancy school to strengthen women’s social interactions.

### Childbirth self-efficacy

The explained variance in FOC increased to 64.5% when we included self-efficacy in the model in the third step. It is clear that childbirth self-efficacy plays an important role in predicting FOC. On the one hand, self-efficacy reflects personal beliefs about behavior that influence outcomes [64]. On the other hand, self-efficacy is the individual’s confidence that they can succeed in performing that behavior in reality [65]. Women with a low level of self-efficacy may exaggerate the difficulty of a natural birth and have lower confidence in their ability to cope with the birth process. Previous studies have reflected that low self-efficacy is connected with severe FOC [47, 66]. Therefore, failing to increase their confidence in their childbirth efficacy may set women up for a distressing birth experience. Research has demonstrated positive outcomes from interventions that may be effective in increasing childbirth confidence, such as pregnancy yoga [67], mindfulness training [68], and antenatal education [69]. A New Zealand study reported that skills-based childbirth preparation contributed to an increase in mothers’ self-efficacy [70]. The program included breathing exercises, verbal and nonverbal communication exercises, tension-reducing exercises, and body exercises as well as advice about stages, delivery methods, and when to use certain skills.

Another interesting finding of this study is that we found a mediating effect of childbirth self-efficacy on the relationship between resilience and FOC. In other words, a better level of resilience brought out stronger self-efficacy, which in turn reduced the CAQ score. The mediation effect rate was 53.5%, confirming that resilience indirectly acted on FOC through self-efficacy. The reason may be that pregnant women with higher resilience make fuller use of their psychological resources to arouse and strengthen their mental capacity to accept the birth event and reevaluate it, and this in turn is beneficial in giving them greater confidence regarding childbirth, thus reducing fear. The mediating effect of childbirth self-efficacy on resilience and FOC found through an SEM provided new insight into those factors influencing FOC. This finding showed that preventive interventions aimed at enhancing resilience and self-efficacy may be conducive to effectively alleviating pregnant women’s fear.

## Conclusions

In sum, we found a high prevalence of FOC among pregnant women in China. Health care professionals should attach importance to FOC and address this issue thoroughly. Age, gestational age, parity, spousal support, resilience and childbirth self-efficacy are predictors of FOC. The findings help us to identify the characteristics of patients with FOC and to formulate corresponding countermeasures. Another point to note is that interventions focusing on enhancing resilience and self-efficacy may alleviate FOC. To provide favorable external conditions for the development of resilience, it is necessary for antenatal care providers to offer timely support to pregnant women through psychological counseling and create peer support groups that allow women to share their fears, experiences and stories and techniques for coping with labor pain. In addition, health care professionals should attach importance to cultivating women’s childbirth self-efficacy via various channels, such as strengthening prenatal education, offering pregnancy yoga courses, and organizing companion-integrated childbirth preparation [24].

## Strengths and limitations

This study has several strengths. First, it has shed light on the relation between resilience and FOC, as there are no relevant studies on this topic. Second, we used an SEM and found a mediating effect of childbirth self-efficacy on the relationship between resilience and FOC, which may provide valuable information for health care professionals.

Regarding limitations, first, this study was conducted in a university-affiliated hospital in a large urban area, which may not allow the current results to represent all pregnant Chinese women. Further research should focus on women in rural and remote communities. Second, because this study was cross-sectional, conclusions about the causal relation between FOC and related factors could not be derived. Thus, future prospective studies are needed.

## Data Availability

The datasets used and/or analysed during the current study are available from the corresponding author on reasonable request.

## Abbreviations

FOC: Fear of childbirth
CAQ: Childbirth Attitude Questionnaire
CBSEI-C32: The short form of 32-item Chinese Childbirth Self-Efficacy Inventory
CD-RISC-10: The 10-item Connor-Davidson Resilience Scale
OE: Outcome Expectancy Subscale
EE: Efficacy Expectancy Subscale
RMB: Renminbi
CVI: Content validity index
SD: Standard deviation
ANOVA: Analysis of variance
DW: Durbin–Watson
VIF: Variance inflation factor
SEM: Structural equation model
CI: Confidence interval
RMSEA: Root mean square error of approximation
GFI: Goodness of fit index
AGFI: Adjusted goodness of fit index
CFI: Comparative fit index
NFI: Normed fit index
IFI: Incremental fit index
RFI: Relative fit index
